# Peripheral BDNF Response to Physical and Cognitive Exercise and Its Association With Cardiorespiratory Fitness in Healthy Older Adults

**DOI:** 10.3389/fphys.2020.01080

**Published:** 2020-08-25

**Authors:** Olga Tarassova, Maria M. Ekblom, Marcus Moberg, Martin Lövdén, Jonna Nilsson

**Affiliations:** ^1^The Swedish School of Sport and Health Sciences, Stockholm, Sweden; ^2^Department of Neuroscience, Karolinska Institutet, Stockholm, Sweden; ^3^Aging Research Center, Karolinska Institutet and Stockholm University, Stockholm, Sweden; ^4^Department of Psychology, Gothenburg University, Gothenburg, Sweden

**Keywords:** plasma BDNF, serum BDNF, cognitive exercise, aerobic exercise, cardiorespiratory fitness, older adults

## Abstract

Physical exercise (PE) has been shown to improve brain function via multiple neurobiological mechanisms promoting neuroplasticity. Cognitive exercise (CE) combined with PE may show an even greater effect on cognitive function. Brain-derived neurotrophic factor (BDNF) is important for neuroplastic signaling, may reduce with increasing age, and is confounded by fitness. The source and physiological role of human peripheral blood BDNF in plasma (pBDNF) is thought to differ from that in serum (sBDNF), and it is not yet known how pBDNF and sBDNF respond to PE and CE. A training intervention study in healthy older adults investigated the effects of acute (35 min) and prolonged (12 weeks, 30 sessions) CE and PE, both alone and in combination, on pBDNF and sBDNF. Cross-sectional associations between baseline pBDNF, sBDNF and cardiorespiratory fitness (CRF) were also investigated. Participants (65–75 years) were randomly assigned to four groups and prescribed either CE plus 35 min of rest (*n* = 21, 52% female); PE [performed on a cycle ergometer at moderate intensity (65–75% of individual maximal heart rate)] plus 35 min of rest (*n* = 27, 56% female); CE plus PE (*n* = 24, 46% female), or PE plus CE (*n* = 25, 52% female). Groups were tested for CRF using a maximal treadmill ergometer test (VO2peak); BDNF levels (collected 48 h after CRF) during baseline, after first exercise (PE or CE) and after second exercise (PE, CE or rest); and cognitive ability pre and post 12-week training. At both pre and post, pBDNF increased after CE and PE (up to 222%), and rest (∼67%), whereas sBDNF increased only after PE (up to 18%) and returned to baseline after rest. Acute but not prolonged PE increased both pBDNF and sBDNF. CE induced acute changes in pBDNF only. Baseline pBDNF was positively associated with baseline sBDNF (*n* = 93, *r* = 0.407, *p* < 0.001). No changes in CRF were found in any of the groups. Baseline CRF did not correlate with baseline BDNF. Even though baseline pBDNF and sBDNF were associated, patterns of changes in pBDNF and sBDNF in response to exercise were explicitly different. Further experimental scrutiny is needed to clarify the biological mechanisms of these results.

## Introduction

A growing body of research and literature reviews indicate that brain function and cognitive performance can be improved in both younger and older adults with physical exercise (PE) (Voss et al., 2011; Chang et al., 2012). These beneficial effects can be accomplished via multiple neurobiological mechanisms that induce and promote neuroplasticity. Cognitive exercise (CE) alone may also induce improvements in cognitive function, however, a combination of physical and cognitive training have been shown to have a greater effect on cognitive performance, as reviewed by Lauenroth et al. (2016). Brain-derived neurotrophic factor (BDNF) has been shown to be essential for neuroplasticity (Poo, 2001; Binder and Scharfman, 2004) and increases in response to PE (Dinoff et al., 2017).

Since the discovery of BDNF (Barde et al., 1982), several studies have reported its significant role in neuronal and synaptic plasticity. It is responsible for long-term potentiation (LTP) in both the developing and mature brain, may regulate learning and memory, and is important for rehabilitation strategies for neurodegenerative and/or neuropsychiatric disease and the regulation of neuropathic pain (Binder and Scharfman, 2004; Park and Poo, 2013). BDNF is expressed in both the central (CNS) and peripheral nervous systems (PNS). It is found in different brain regions, cerebrospinal fluid, neural and non-neural cells and peripheral tissue, and in blood (Barde et al., 1982; Donovan et al., 1995; Rosenfeld et al., 1995; Radka et al., 1996; Nakahashi et al., 2000; Matthews et al., 2009). A study in drug-naïve first-episode psychotic patients reported a significant positive moderate correlation (*r* = 0.509, *p* = 0.03) between BDNF levels in plasma and cerebrospinal fluid (Pillai et al., 2010). It is also known that blood vessels of the CNS possess a unique blood–brain barrier (BBB) property that allow the regulation of movement of different cells between the blood and the brain (Abbott et al., 2010; Daneman and Prat, 2015; Keaney and Campbell, 2015). Moreover, it has been shown that BDNF is possibly able to bi-directionally cross animal (Pan et al., 1998) and human BBB, from brain to blood (Krabbe et al., 2007). The latter may indicate that an increase in peripheral BDNF could be a result of elevated secretion of central BDNF in humans. However, more studies are required to support this hypothesis. In blood, BDNF is mainly stored in thrombocytes, also called platelets, which when activated release BDNF into the blood. Blood plasma contains much lower levels of circulating (not stored in platelets) BDNF that is hypothesized to have a different physiological role to the platelet-bound BDNF, which can be measured in blood serum (Fujimura et al., 2002; Lommatzsch et al., 2005). In human *in vivo* studies, the only feasible way to measure BDNF level changes is by measuring peripheral BDNF in blood plasma or serum.

Inconsistent results have been shown concerning the association between peripheral plasma BDNF (pBDNF) and serum BDNF (sBDNF) in humans. For example, two studies found no correlation between baseline BDNF levels in plasma and serum (Bocchio-Chiavetto et al., 2010; Tsuchimine et al., 2014), whereas other studies have found a positive weak (*r* = 0.349, *p* < 0.05) (Kudlek Mikulic et al., 2017), moderate (*r* = 0.596, *p* < 0.01) (Polyakova et al., 2017) or even strong correlation (*r* = 0.731, *p* < 0.0001) (Yoshimura et al., 2010). This discrepancy may be caused by the different populations and experimental conditions used between studies.

Peripheral BDNF concentrations have been shown to decrease with age (Lommatzsch et al., 2005; Ziegenhorn et al., 2007). Since BDNF plays a significant role in neuroplasticity, an age-related decline in BDNF may reduce neuroplastic signaling in older adults. It is therefore of great interest to further our understanding of how CE and PE interventions aimed to promote neuroplastic signaling influence levels of pBDNF and sBDNF in older adults.

Even though results from previous studies are somewhat inconsistent it has been shown that acute PE can induce a transient increase in pBDNF and sBDNF (Knaepen et al., 2010; Szuhany et al., 2015; Dinoff et al., 2017). Dinoff et al. (2017) reported that acute exercise for 7–240 min at 45–100% of maximal oxygen uptake can cause up to a 40% increase in peripheral BDNF in young adults, with a greater increase found in plasma compared to serum. Moreover, a physical training with prescribed exercise of 20–90 min for 2–7 sessions per week for 5–52 weeks was also shown to increase BDNF concentrations in peripheral blood in young adults (Dinoff et al., 2016). In older adults, acute and/or chronic aerobic exercise has been shown to affect both pBDNF and sBDNF (Coelho et al., 2013; Håkansson et al., 2017). Interestingly, in a seminal paper, Erickson et al. (2011) showed that higher levels of sBDNF after exercise were associated with an increased hippocampal volume, which in turn was associated with improved memory performance. However, to our knowledge, only one recent study has investigated the effects of exercise on both pBDNF and sBDNF in older adults, and the authors found an increase in sBDNF but not in pBDNF in response to acute exercise (Máderová et al., 2019).

There is still insufficient evidence on whether acute CE or prolonged cognitive training alone increases peripheral BDNF concentrations in healthy older adults. For example, no significant changes in sBDNF were found after acute CE in healthy older adults (Håkansson et al., 2017), or after 10 weeks of cognitive training in a group consisting mostly of older adults with mild cognitive impairment (Küster et al., 2017). Ledreux et al. (2019) did find an increase after 5 weeks of cognitive training in healthy older adults, but only in their Swedish and not their American cohort. It is unclear why.

Several studies have addressed the importance of investigating the relationship between aerobic fitness and BDNFs (Nofuji et al., 2008; Currie et al., 2009; Jung et al., 2011; Hwang et al., 2017). The nature of this relationship may further clarify the physiological regulation of peripheral BDNF in response to regular exercise (Currie et al., 2009), and the beneficial effects of aerobic fitness on cognitive function (Hwang et al., 2017). Hwang et al. (2017) showed BDNF positively moderated a positive relationship between aerobic fitness and working memory performance but only in those subjects with the highest VO_2_max levels. Moreover, Currie et al. (2009) suggested that the inverse association between baseline cardiorespiratory fitness (CRF) and sBDNF reported in previous studies (Currie et al., 2009; Jung et al., 2011; Hwang et al., 2017) can possibly be explained by changes in physiological control of platelet mobilization and function in response to regular exercise (Currie et al., 2009). Studies reviewed in Dinoff et al. (2017) showed that participants with greater CRF show greater increases in peripheral BDNF after acute PE, suggesting that individuals with greater fitness could be more adapted to the acute physiological changes (Dinoff et al., 2017). However, the majority of previous studies investigating the relationship between BDNF and CRF mainly included only younger adults, and assessed only serum BDNF. Therefore, in older adults, a relationship between CRF and changes in both plasma and serum BDNF should also be considered while investigating the potential effects of training on peripheral BDNF.

The primary aim of the present study was to investigate how pBDNF and sBDNF respond to PE and CE, both acute and after a 12-week training period, in older adults. Secondary aims were to describe how baseline pBDNF and sBDNF are related to each other and to CRF, and how changes in pBDNF, sBDNF and CRF over the intervention period are interrelated.

## Materials and Methods

### Participants

Via advertisements in local media, 139 healthy individuals aged between 65 and 75 years, free from any serious physiological or psychological illness and trauma, were recruited for this study. The study inclusion criteria is described in more detail in supplementary material in the previous study (Nilsson et al., 2020). Participant suitability for the fitness tests and training was confirmed via a telephone interview with a medical professional and an initial information meeting. This study was approved by the Ethical Review Board in Stockholm (2017/1115-31/4) and conducted in accordance with the Declaration of Helsinki. A written informed consent was obtained from all participants.

### Experimental Protocol

#### Overview

**FIGURE 1:**
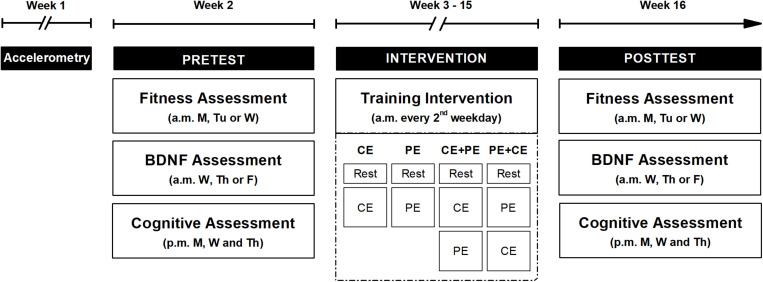
An overview of the study design. The study procedure included three parts: pretest (Pretest), a 12-week training period (Intervention) and posttest (Posttest). Both pre- and posttests were preceded with 1 week of participant physical activity level measurements (Accelerometry) using a lightweight triaxial accelerometer worn on the hip during the day. However, physical activity measurements during the final week of training period are not depicted in the figure. A cardiorespiratory fitness test (Fitness Assessment) and an assessment of acute changes in BDNF concentration (BDNF Assessment) were performed in the morning (a.m.) for both pre- and posttests. The fitness assessment was performed 48 h prior to the BDNF assessment. During the week of pre- and posttest, 3 afternoon (p.m.) sessions included a cognitive assessment (Cognitive Assessment). During the intervention period, participants performed a training session every second weekday of either: cognitive exercise only (CE), physical exercise only (PE), cognitive exercise followed by physical exercise (CE + PE) or physical exercise followed by cognitive exercise (PE + CE), according to their allocated intervention. Each training visit started with 15 min of seated rest (Rest). M, Monday; Tu, Tuesday; W, Wednesday; Th, Thursday; F, Friday.

The study procedure consisted of: (1) physical activity measurements, (2) pretest sessions, (3) 12-week training period with physical activity measurements conducted during the final week of training period, and (4) posttest sessions (Figure 1). Each participant recruited for this study was randomly assigned to one out of four training groups, which were prescribed either 35 min of CE followed by 35 min of rest (CE); 35 min of PE followed by 35 min of rest (PE); the CE followed by the PE (CE + PE), or the PE followed by the CE (PE + CE). Participant age, general cognitive performance score (reasoning task, score range 0–18) and self-reported physical activity level (score range 0–5) assessed during the initial screening were used as stratifiers for group randomization. The randomization procedure was performed using the R Programming Environment (version 3.5.129) and was conducted in separate rounds of approximately 24–32 participants using label shuffling with *post hoc* non-parametric tests for the stratifiers, as described in a previous study that investigated the same population (Nilsson et al., 2020). During the entire study period, participants were asked to refrain from: (a) making major lifestyle changes in exercise routine, diet or hobbies, (b) drinking coffee, tea or any other caffeinated drink 2 h before each study visit, and (c) excessive amounts of alcohol and PE of very high intensity.

#### Pretest and Posttest Sessions

Physical activity patterns were assessed for 7 days prior to the pretest sessions using a lightweight accelerometer worn by the participant on an elastic band on the hip during the day. The pretest sessions included, a CRF assessment, a BDNF assessment (48 h after the CRF assessment) and 3 afternoon sessions of cognitive assessment (Figure 1).

The posttest sessions were completed in the week following the end of the 12-week training period, in the same order as the pretest sessions. Physical activity patterns were assessed for 7 days prior to the posttest sessions, during the final week of the training period. The posttest CRF and BDNF assessment were performed in the same way as in the pretest session, but participants included in the CE, CE + PE and PE + CE groups performed the CE with a higher level of difficulty than in the pretest, i.e., each participant continued from the same level of difficulty individually achieved in the final cognitive training session of the training period.

Participant height and weight were measured at both pre- and posttest, prior to the CRF assessment, and used for calculation of Body Mass Index (BMI). CRF and BDNF assessments were always scheduled in the first half of the day (08:00–12:30 a.m.) to avoid variability due to individual diurnal fluctuations in the obtained measures. On the day of each study visit involving BDNF assessments, participants were asked to consume the same type and size of breakfast. Each BDNF assessment session started with 15 min of seated rest in order to limit previous physical activity as a potential source of variation in baseline BDNF concentrations.

#### Training Period and Exercise Protocols

##### Training period

The training period included a maximum of 32 training sessions that were scheduled for each participant every second weekday during a 12-week period (i.e., 2–3 sessions per week on alternating weeks) (Figure 1). The duration of the training period and the number of sessions per week were chosen based on previous studies on working memory performance (Nilsson et al., 2017; Lebedev et al., 2018) and on the effects of physical and cognitive training on cognition in older adults (Gheysen et al., 2018). The feasibility of the duration and intensity of the intervention in an older population was also taken into consideration. The participant performed each training session at the same time of the day (between 08:00 and 12:30 a.m.). The sessions included an initial 15 min of seated rest followed by the type of exercise session chosen according to the intervention group the participant was assigned to (CE, PE, CE + PE or PE + CE) (Figure 1).

##### Physical exercise

Physical exercise (∼35 min) was performed on a cycle ergometer (model 828E, Monark, Sweden) during the BDNF assessments at pre- and posttest, and during the 12-week training period by PE, CE + PE and PE + CE groups. After an initial 5-min warm-up at around 65% of the participant’s maximal heart rate (HRmax), participants cycled for 30 min at a varied (every 5th min) heart rate (HR): 65%, 70%, 75%, 70%, 75%, and 65% of the participant’s HRmax. Participants were advised to cycle at a cadence of around 70 revolutions per minute (rpm). HR levels were achieved by manipulating the resistance of the cycle ergometer. HR, measured using a heart rate sensor (worn on a chest strap) and Polar monitor (m400, Polar Electro Oy, Kempele, Finland), was displayed to the participants for continuous self-monitoring. To ensure that the exercise intensity would not be too low and dictated largely by the leg strength rather than aerobic capacity, in this study of older adults, the HRmax was measured during the maximal treadmill ergometer test (see section “Fitness Assessment”) rather than in cycling. The Rated Perceived Exertion (RPE) according to the Borg scale (Borg, 1970), was monitored throughout the whole PE session and exercise intensity was adjusted accordingly if the RPE fell outside the specified range of 13–16. Since cycle ergometer resistance was regulated based on HR, the ergometer resistance used during the posttest was higher than the resistance used during the pretest. To ensure participant safety, PE intensity, which was administered by trained personnel with appropriate cardiopulmonary resuscitation (CPR) training, was restricted to 16 RPE or under. Trained personnel also checked health status of the participant prior to each PE session.

##### Cognitive exercise

Cognitive exercise (∼35 min) was performed during the BDNF assessments at pre- and posttest, and during the 12-week training period by CE, CE + PE and PE + CE groups. Protocol mainly focused on the working memory construct of updating. Updating is the ability to continuously maintain and update mental representations trained using *n*-back and running-span tasks. For the running-span task, the participant has to remember the 2 or 3 latest presented stimuli in a series of stimuli shown to them. For the *n*-back task, the participant has to detect if the current stimulus is the same as the one presented 2 or 3 steps back. All tasks were designed with different levels of difficulty (adaptive tasks) and each level was defined by a fixed criterion (e.g., certain number of correct responses) that the participant had to reach in order to proceed to the next level. Such a progressive increase in task difficulty is hypothesized to be important for inducing neuroplasticity (Lövdén et al., 2010). In the last 5 min of each cognitive session, each participant performed a non-adaptive (identical across the whole study period) *n*-back test used as a measure of learning due to cognitive training. The participant’s progress measures were presented on the screen as cartoon figures including scores and a process indicator figure. The latter was expected to increase the participant’s motivation to improve during the training period.

### Physical Activity Measurements: Accelerometer

Physical activity measurements were used as a quality control to ensure that the participants did not compensate their average physical activity pattern by replacing their usual exercise habits with the exercise used in the intervention. Accelerometer measurements were performed in order to assess the participants’ usual physical activity outside the procedures of the present study and to capture any potential changes in physical activity before and in the final week of the 12-week training period. For 1 week (7 days) prior to both the pretest and posttest, participants wore a lightweight accelerometer (Actigraph GT3X+, Actigraph LCC; Pensacola, FL, United States) attached to an elastic band and placed on the hip during the day (06:00–23:00). Raw triaxial accelerometer data were recorded at a sample rate of 30 Hz. A resulting vector was extracted as 60 s epoch using a low frequency extension filter. A minimum of 600 min of valid daily wear time for at least 4 days was required to be included in the analysis (Trost et al., 2005). Sedentary behavior was defined as <200 counts per minute (cpm) and moderate-to-vigorous physical activity as cpm ≥ 2690 (Sasaki et al., 2011). Proportions of time between 06:00 and 23:00 spent sedentary (SED) and in moderate-to-vigorous physical activity (MVPA) was expressed in relative (%) units and used for further analysis.

### Fitness Assessment

Maximal treadmill ergometer test, which was conducted by trained personnel with CPR training, allowed direct measurement of peak rate of oxygen consumption (VO2peak) and HRmax. An initial 5–10 min warm-up session prior to the maximal test allowed the participants to familiarize themselves with the equipment. The warm-up session was followed by the maximal test. Each participant wore a harness attached to the ceiling to protect from falling. HR was measured using the same sensor as during the intervention. The maximal test started with a treadmill incline of 1 degree and an individually selected comfortable speed (around 12–13 RPE). This was increased every minute until volitional exhaustion. VO2peak was measured using a computerized metabolic system (Jaeger Oxycon Pro, Hoechberg, Germany). Similarly to Björkman et al. (2016), the VO2peak measurement was accepted if a minimum of three out of five following criteria were achieved: (a) VO_2_ was leveling off despite an increase in speed or decline, (b) RPE reached a value above 16, (c) a respiratory exchange ratio reached value greater than 1.1, (d) HRmax within ±15 beats per minute (bpm) from age-predicted HRmax, and (e) a work with time above 6 min was performed. The highest 30 s registered values of VO_2_ and HR were referred to as VO2peak and HRmax, respectively. The directly measured VO2peak used for further analysis were expressed in relative units (mL kg^–1^ min^–1^).

### Cognitive Assessment

Cognitive assessment session consisted of 19 tests and was conducted in the afternoon during both pre- and posttest in order to assess the effect of cognitive and physical training on the participants’ cognitive performance. This assessment aimed to measure eight cognitive constructs and included trained working memory tasks (trained stimuli and untrained stimuli), untrained working memory tasks (updating, switching) and untrained cognitive domains (episodic memory, processing speed, spatial reasoning, and verbal reasoning). The results of this assessment, however, were not an aim and main outcome of the present study and together with a detailed description of the cognitive assessment protocol are presented elsewhere (Nilsson et al., 2020).

### Blood Sampling and BDNF Assessment

**FIGURE 2:**
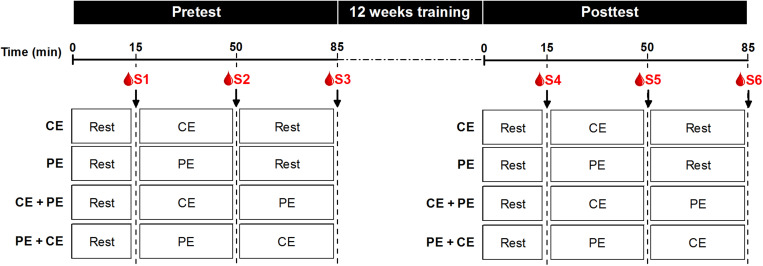
BDNF assessment protocol at both pre- and posttest. Both pre- and posttest sessions started with 15 min of seated rest (Rest). Thereafter participants performed a training session of either: cognitive exercise only (CE) followed by 35 min of rest, physical exercise only (PE) followed by 35 min of seated rest, cognitive exercise followed by physical exercise (CE + PE) or physical exercise followed by cognitive exercise (PE + CE), according to their allocated intervention. Blood samples were drawn immediately after 15 min of rest (S1 and S4 at pretest and posttest, respectively), immediately after the first exercise (S2 and S5 at pretest and posttest, respectively) and immediately after the second exercise or rest (S3 and S6 at pretest and posttest, respectively).

Brain-derived neurotrophic factor assessment session was scheduled for each participant at the same time of day (08:00–12:30 a.m.) at both pre- and posttest to avoid variability due to individual diurnal fluctuations in BDNF levels. For both pre- and posttest, each session started with a catheter being inserted into the participant’s antecubital vein by a medical professional. Thereafter, the following procedure was conducted: 15 min of seated rest, the first blood sample (baseline, termed S1 and S4 for pre- and posttest, respectively), the first exercise session (either CE or PE depending on the assigned intervention), the second blood sample (termed S2 and S5 for pre- and posttest, respectively), the second exercise session (either CE or PE or 35 min of seated rest depending on the assigned intervention) and the third blood sample (termed S3 and S6 for pre- and posttest, respectively) (Figure 2). The catheter was flushed with saline solution between each blood sampling to avoid contamination between samples. Blood sampling was conducted by appropriately trained personnel and the proximity of a medical professional in case of emergency was ensured at all times. In total, six blood samples were collected [three at pretest (S1, S2, and S3) and three at posttest (S4, S5, and S6)]. For each blood sample, approximately 10 mL blood was collected into two separate containers: a heparinized tube for plasma and a clot activator tube for serum analysis. Directly after the blood was drawn, the tube containing blood for plasma analysis was centrifuged at 6000 rpm for 3 min to separate the plasma. The tube for serum analysis was left to clot at room temperature for at least 30 min, after which it was centrifuged at 6000 rpm for 15 min to separate the serum. All centrifuging was performed at 4°C. Thereafter, both separated plasma and serum samples were transferred to Eppendorf tubes and stored at −80°C until analysis.

For the quantification of BDNF concentrations, both serum and plasma samples were analyzed in duplicate using the Quantikine Human Free BDNF Immunoassay kit (ELISA, DBD00, R&D Systems, Inc., Minneapolis, MN, United States) and the manufacturer’s instructions. Serum samples were diluted 20x prior to analysis in the supplied assay buffer. The BDNF concentrations measured and used for analysis were expressed in pg/mL. Intra-assay variability for both plasma and serum ranged between 2.6 and 3.6% for all assays completed. We also determined inter-assay variability to be 10.4%.

### Statistical Analysis

All analyses were performed using the R Programming Environment (version 3.6.2). All comparisons using analysis of variance (ANOVA) were performed with type III ANOVA using the *afex* package (version 0.26.0) with Mauchly’s Test of Sphericity used to examine a violation of the assumption of sphericity. If this assumption was violated, a Greenhouse–Geisser correction was applied. *Post hoc* comparisons of estimated marginal means were performed using the *emmeans* package (version 1.4.4) with adjustment for multiple comparisons according to the Tukey method.

A Shapiro–Wilk test was used to assess the normal distribution of all continuous variables. All analyzed demographic variables including physical activity measures, CRF and sBDNF showed an approximate normal distribution in all of investigated groups and conditions. However, pBDNF deviated substantially from the normal distribution. Natural-log-transformed (ln) pBDNF (ln-pBDNF) values were therefore used for all statistical analyses for pBDNF. All normally distributed variables were expressed as mean ± standard deviation (SD) or ± standard error (SE). Variables that deviated from the normal distribution were expressed as back-transformed [e^*ln(x*)^] natural-log mean values with 95% upper and 95% lower limits as variance estimates.

Separate mixed-design ANOVAs with one between-subject factor “*group*” (CE, PE, CE + PE, and PE + CE) and one within-subject time factor “*prepost*” (pretest, posttest) were used to assess changes from pre- to posttests for BMI, VO2peak, SED and MVPA, and differences between groups.

Separate mixed-design ANOVAs with three factors: one between-subject factor “*group*” and two within-subject time factors “*prepost*” (pretest, posttest) and “*time*” [baseline (S1 or S4), acutely after first exercise (S2 or S5), acutely after second exercise or rest (S3 or S6)] were performed to compare ln-pBDNF and sBDNF concentrations between groups and conditions.

Pearson product-moment correlation coefficients (*r*) were calculated to assess the relationships between ln-pBDNF and sBDNF measured at baseline (ln-pBDNF_*S*1_ versus sBDNF_*S*1_), and between baseline CRF and baseline BDNF (VO2peak_*pre*_ versus ln-pBDNF_*S*1_ and sBDNF_*S*1_).

Acute changes in non-transformed pBDNF and sBDNF were calculated as differences between concentrations measured at pretest, acutely after the first exercise (S2), and at baseline (S1) denoted as pBDNF_*S*2–*S*1_ and sBDNF_*S*2–*S*1_. Long-term changes in BDNF and CRF were calculated as differences between values measured at posttest baseline (S4) and pretest baseline (S1) for non-transformed pBDNF and sBDNF (pBDNF_*S*4–*S*1_ and sBDNF_*S*4–*S*1_), and as posttest–pretest differences for VO2peak (VO2peak_*post*–*pre*_).

As many of the above calculated changes deviated from normal distribution, non-parametric Kendall’s tau-b (τ_*b*_) correlation coefficients were calculated in either the whole sample, separately in each of the investigated groups or both to assess the following relationships: pBDNF_*S*4–*S*1_ versus sBDNF_*S*4–*S*1_, and VO2peak_*post*–*pre*_ versus pBDNF_*S*4–*S*1_ and sBDNF_*S*4–*S*1_. Kendall’s tau-b correlation coefficients were also calculated to assess the relationship between pBDNF_*S*2–*S*1_ and sBDNF_*S*2–*S*1_, however, for COG (CE and CE + PE groups combined) and PHYS (PE and PE + CE groups combined) groups.

The level of significance was set at *p* < 0.05.

## Results

### Descriptive

Of the 139 participants, 42 dropped out either before the start or during the study due to various disclosed or undisclosed reasons, including illness. A recruitment diagram with a detailed description of the drop-outs and reasoning are described in more detail in the supplementary material from Nilsson et al. (2020). Of the 97 participants who completed the study, there were incomplete data for four participants due to being an outlier at either pre- or posttest, or technical issues, and incomplete data for six participants at posttest due to either sickness or because one or more inclusion criteria were no longer met.

The participants’ demographic and physiological descriptions are presented in Table 1. Results from the mixed-design ANOVAs with one between-subject factor “*group*” (CE, PE, CE + PE, and PE + CE) and one within-subject time factor “*prepost*” (pretest and posttest) showed no significant overall differences (main effect of “*group*”) between the investigated groups (CE, PE, CE + PE, and PE + CE) for BMI [*F*(3, 88) = 0.75, *p* = 0.53], VO2peak [*F*(3, 86) = 0.74, *p* = 0.53], SED [*F*(3, 86) = 0.20, *p* = 0.90] and MVPA [*F*(3, 86) = 0.99, *p* = 0.40]. No significant main effect of “*prepost*” or interaction “*group* × *prepost*” was found for the above variables except for SED. Participants showed an overall increase in time spent sedentary [*F*(1, 86) = 13.11, *p* < 0.001] in the final week of the training period (mean = 58.3%, SE = 0.9%) compared to the week before the pretest (mean = 56.0%, SE = 0.9%).

**TABLE 1:** Group descriptives.

	CE			PE			CE + PE			PE + CE		
	Mean	SD	*n*	Mean	SD	*n*	Mean	SD	*n*	Mean	SD	*n*
**Age (years)**												
Pretest	71.0	3.0	21	70.3	3.0	27	70.3	3.1	24	70.3	2.7	25
**Sex (female/male, %)**												
Pretest	52/48	−	21	56/44	−	27	46/54	−	24	52/48	−	25
**Height (cm)**												
Pretest	173.5	9.4	20	171.8	9.1	25	171.7	6.5	22	169.3	9.8	25
**Weight (kg)**												
Pretest	76.3	12.9	21	77.5	12.6	27	76.2	11.7	24	71.9	14.4	25
Posttest	76.2	12.5	21	77.7	12.4	27	75.6	11.6	24	71.3	15.0	24
**BMI (kg/m^2^)**												
Pretest	25.7	3.5	20	26.2	3.2	25	26.0	2.6	22	25.0	3.9	25
Posttest	25.6	3.4	20	26.3	3.2	25	25.8	2.6	22	24.8	4.1	24
**VO2peak (mL kg**^–^**^1^ min**^–^**^1^)**												
Pretest	31.0	5.0	21	30.9	5.0	26	31.0	6.0	23	33.1	5.7	25
Posttest	30.7	5.3	21	30.4	4.7	25	31.8	5.2	21	32.7	5.9	23
**SED (%)**												
Pretest	56.6	10.4	20	55.3	9.0	27	56.2	8.4	23	55.8	6.4	25
Posttest	58.8	8.6	17	59.5	8.0	26	58.7	8.1	23	56.5	8.0	24
**MVPA (%)**												
Pretest	6.2	2.7	20	6.3	1.6	27	6.2	2.5	23	6.8	3.2	25
Posttest	5.6	2.2	17	6.3	2.5	26	6.3	2.4	23	7.1	2.9	24

*Mean (mean) ± standard deviation (SD) of age, height, weight, body mass index (BMI), sex expressed as % female/male, cardiorespiratory fitness measured using maximal treadmill test (VO2peak), time spent sedentary (SED) (%) and time spent in moderate-to-vigorous physical activity (MVPA) (%) for each of the investigated groups at pretest (pretest) and posttest, i.e., after a 12-week training period (posttest). CE, group received cognitive exercise only; PE, group received physical exercise only; CE + PE, group received cognitive followed by physical exercise; PE + CE, group received physical followed by cognitive exercise; n, number of participants.*

### Plasma and Serum BDNF Response to Cognitive and Physical Exercise

Descriptive statistics of non-transformed pBDNF and sBDNF measured in samples taken at pretest (S1, S2, and S3) and posttest (S4, S5, and S6) for each of the investigated groups (CE, PE, CE + PE, and PE + CE) are presented in Supplementary Table 1.

Table 2 displays the results of the investigated main and interaction effects in the comparisons of ln-pBDNF and sBDNF using mixed-design ANOVA with three factors: “*group*,” “*prepost*” and “*time*” [for pretest: baseline (S1), acutely after first exercise (S2), acutely after second exercise or rest (S3) and for posttest: baseline (S4), acutely after first exercise (S5), acutely after second exercise or rest (S6)].

**TABLE 2:** Comparisons of natural-log-transformed plasma BDNF (ln-pBDNF) and serum BDNF (sBDNF) between groups and conditions.

	ln-pBDNF				sBDNF			
Factors	*df*_factor_	*df*_error_	*F*	*p*	*df*_factor_	*df*_error_	*F*	*p*
Group	3.00	85.00	0.22	0.88	3.00	85.00	1.86	0.14
Prepost	1.00	85.00	14.30	**<*0.001***	1.00	85.00	1.28	0.26
Time	1.83	155.91	207.47	**<*0.001***	1.79	151.80	38.36	**<*0.001***
Group × prepost	3.00	85.00	3.32	***0.02***	3.00	85.00	0.27	0.84
Group × time	5.50	155.91	1.01	0.42	5.36	151.80	25.92	**<*0.001***
Prepost × time	1.71	145.52	9.02	**<*0.001***	1.57	133.41	2.13	0.13
Group × prepost × time	5.14	145.52	1.42	0.22	4.71	133.41	0.60	0.69

*Results of the investigated main and interaction effects in three-way mixed model ANOVAs with three factors: “group” (CE, PE, CE + PE, PE + CE), “prepost” (pretest and posttest) and “time” [baseline (S1 or S4), acutely after first exercise (S2 or S5), acutely after second exercise or rest (S3 or S6)]. BDNF, brain-derived neurotrophic factor; df_factor_, degrees of freedom for the investigated factor; df_error_, degrees of freedom for the error term of the investigated factor. The level of significance was set at p < 0.05. Significant effects are in bold italics.*

#### Plasma BDNF

**FIGURE 3:**
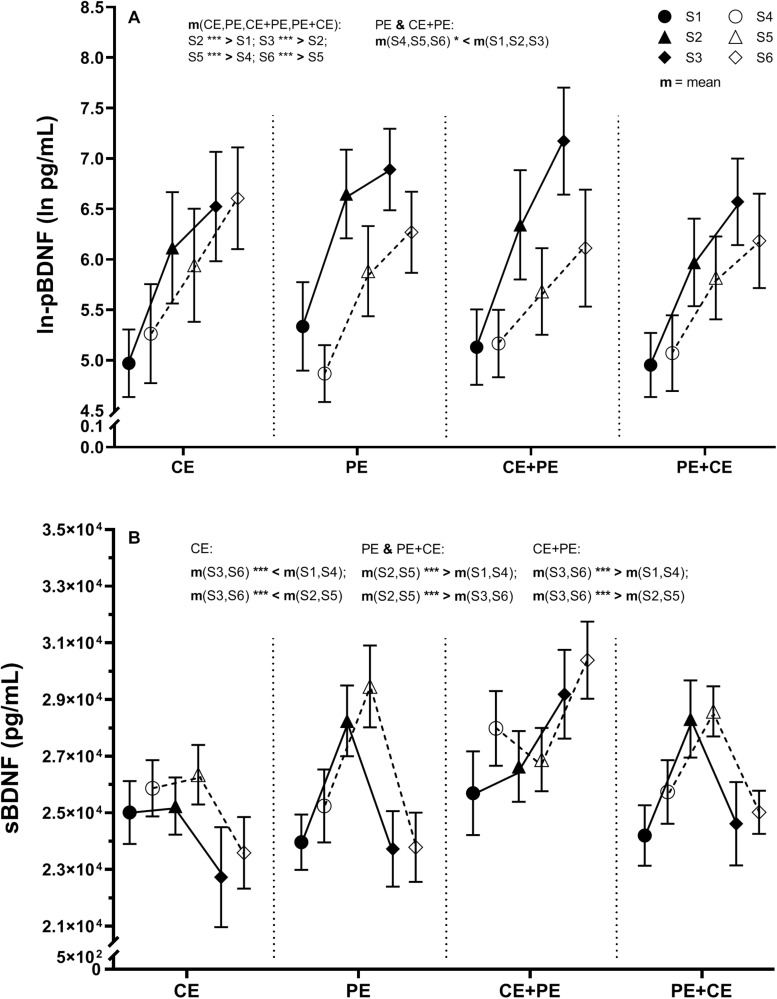
Descriptive data and results from the statistical analysis of **(A)** natural-log transformed plasma BDNF (ln-pBDNF) and **(B)** serum BDNF (sBDNF) response to cognitive and physical exercise. BDNF concentrations were measured in blood samples drawn immediately after 15 min of rest (S1 and S4 at pretest and posttest, respectively), immediately after the first exercise (S2 and S5 at pretest and posttest, respectively) and immediately after the second exercise or rest (S3 and S6 at pretest and posttest, respectively). The investigated groups performed either cognitive exercise only, followed by rest (CE), physical exercise only followed by rest (PE), cognitive followed by physical exercise (CE + PE) or physical followed by cognitive exercise (PE + CE). For ln-pBDNF, values are presented as natural-log means ± 95% CI and for sBDNF as means ± SE. Graphs also display the results from mixed model ANOVA analysis of ln-pBDNF for the significant interaction “*prepost* × *group*” and interaction “*prepost* × *time*,” and of sBDNF for the significant interaction “*time* × *group.*” m, mean; *p < 0.05; ***p < 0.001. The level of significance was set at *p* < 0.05.

There was no significant three-way interaction “*group* × *prepost* × *time.*” However, a significant interaction “*group* × *prepost*” [*F*(3, 85) = 3.32, *p* = 0.02] and “*prepost* × *time*” [*F*(1.71, 145.52) = 9.02, *p* < 0.001] was found for ln-pBDNF (Table 2). Mean changes in ln-pBDNF concentrations including the main results of pairwise comparisons between groups and conditions are presented in Figure 3A. All investigated groups showed a significant (*p* < 0.001) average increase in ln-pBDNF from S1 to S2 [222% average increase in back-transformed natural-log-transformed mean (ln-mean)], and S2 to S3 (67% average increase in back-transformed ln-mean) at pretest, from sample S4 to S5 (101% average increase in back-transformed ln-mean), and S5 to S6 (67% average increase in back-transformed ln-mean) at posttest (Figure 3A). However, a significant (*p* < 0.05) average decrease in ln-pBDNF from pretest to posttest was shown in the PE group (47.8% average decrease in back-transformed ln-mean) and the CE + PE group (48.3% average decrease in back-transformed ln-mean) (Figure 3A).

#### Serum BDNF

A significant effect was only shown for the factor “*time*” [*F*(1.79, 151.80) = 38.36, *p* < 0.001] and an interaction of “*group* × *time*” [*F*(5.36, 151.80) = 25.92, *p* < 0.001] (Table 2). Mean changes in sBDNF including the main results of pairwise comparisons for sBDNF are presented in Figure 3B. Similarly, at both pretest and posttest, groups showed the following significant changes: CE had significantly lower levels of sBDNF after 35 min of rest compared to baseline (9% average decrease, *p* < 0.01), and compared to after CE (10% average decrease, *p* < 0.001); PE had a significant (*p* < 0.001) 18% average increase in sBDNF after PE compared to baseline, which then return to baseline levels after 35 min of rest (18% average decrease, *p* < 0.001); CE + PE had significantly (*p* < 0.001) higher levels of sBDNF after PE compared to baseline (11% average increase), and compared to after CE (12% average increase); PE + CE had a significant (*p* < 0.001) 14% average increase in sBDNF after PE compared to baseline, and then a return to baseline levels after CE (13% average decrease, *p* < 0.001) (Figure 3B).

### Association Between Baseline (and Changes in) Plasma and Serum BDNF

**FIGURE 4:**
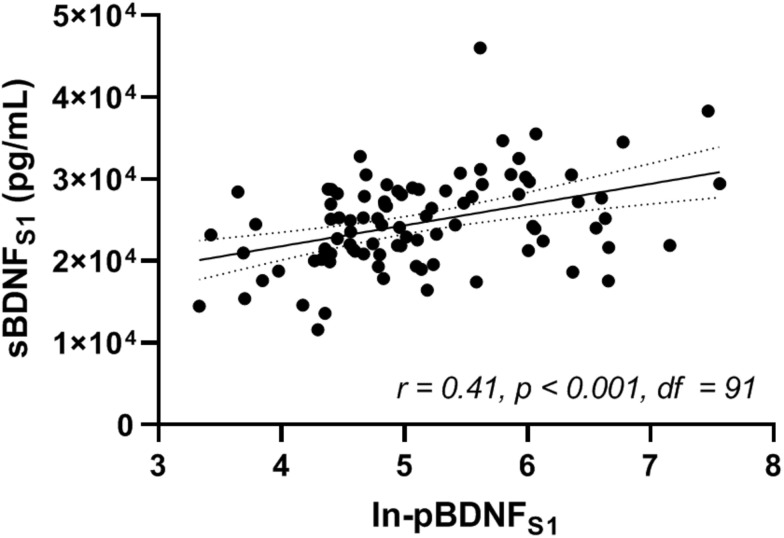
Correlation between natural-log-transformed plasma and serum BDNF (ln-pBDNF_*S*1_ and sBDNF_*S*1_, respectively) measured in blood samples drawn immediately after 15 min of rest at pretest (S1). Analysis was performed for the whole sample using Pearson product-moment correlation coefficient *(r)* and its *p*-value. BDNF, brain-derived neurotrophic factor. *df*, degrees of freedom of the test statistic. The level of significance was set at *p* < 0.05.

Pearson product-moment correlation coefficient was calculated between baseline ln-pBDNF and sBDNF measured at pretest (ln-pBDNF_*S*1_ and sBDNF_*S*1_, respectively) (Figure 4). A significant (*p* < 0.001) weak positive (*r* = 0.41) association was found between ln-pBDNF_*S*1_ and sBDNF_*S*1_ concentrations.

### Association Between Changes in Plasma and Changes in Serum BDNF

No significant association calculated using Kendall’s tau-b (τ_*b*_) correlation coefficient was found between changes, either acute (S2–S1) or in response to 12 weeks of training (S4–S1), in non-transformed pBDNF and sBDNF (Table 3).

**TABLE 3:** Association between changes, both acute (S2–S1) and changes due to training (S4–S1), in plasma BDNF (pBDNF_*S*2–*S*1_ or pBDNF_*S*4–*S*1_, respectively) and in serum BDNF (sBDNF_*S*2–*S*1_ or sBDNF_*S*4–*S*1_, respectively).

	*n*_*pairs*_	τ_*b*_	*p*
**Acute changes**			
**pBDNF_*S*2–*S*1_*vs.* sBDNF_*S*2–*S*1_**			
COG	42	–0.12	0.26
PHYS	51	0.12	0.20
**Changes due to training**			
**pBDNF_*S*4–*S*1_*vs.* sBDNF_*S*4–*S*1_**			
CE	20	–0.13	0.46
PE	25	–0.21	0.16
CE + PE	22	0.01	0.96
PE + CE	25	–0.15	0.32

*Separate analysis were performed for acute changes in groups COG (CE and CE + PE groups combined) and PHYS (PE and PE + CE groups combined), and for changes due to training in groups CE, PE, CE + PE, and PE + CE. Kendall’s tau-b (τ_b_) correlation coefficients and its p-values were calculated. BDNF, brain-derived neurotrophic factor; n_pairs_, number of pairs compared. The level of significance was set at p < 0.05.*

### Association Between Cardiorespiratory Fitness and BDNF

Baseline CRF measured at pretest, VO2peak_*pre*_ (mean = 31.5, SD = 5.5), did not correlate with ln-pBDNF_*S*1_ [*r*(92) = 0.17, *p* = 0.10] or sBDNF_*S*1_ [*r*(90) = 0.06, *p* = 0.56].

There was also no significant correlation between changes (post – pre 12 weeks of training) in CRF (VO2peak_*post*–*pre*_) or changes in baseline BDNF (non-transformed pBDNF_*S*4–*S*1_ or sBDNF_*S*4–*S*1_), except between VO2peak_*post*–*pre*_ and sBDNF_*S*4–*S*1_ in the CE + PE group (τ_*b*_ = 0.37, *p* = 0.02) (Table 4).

**TABLE 4:** Association between changes due to training in baseline cardiorespiratory fitness (VO2peak_*post*–*pre*_) and changes in baseline plasma BDNF or serum BDNF (pBDNF_*S*4–*S*1_ or sBDNF_*S*4–*S*1_, respectively) in the whole sample (ALL) and separately in groups CE, PE, CE + PE, and PE + CE.

	pBDNF_*S4*–*S1*_			sBDNF_*S4*–*S1*_		
	*n*_*pairs*_	τ_*b*_	*p*	*n*_*pairs*_	τ_*b*_	*p*
**VO2peak_*post*–*pre*_**						
ALL	92	0.11	0.882	91	0.12	0.10
CE	21	–0.09	0.61	20	0.07	0.68
PE	25	0.11	0.47	25	–0.03	0.87
CE + PE	22	0.19	0.24	22	***0.37***	***0.02***
PE + CE	24	–0.20	0.17	24	0.04	0.83

*Kendall’s tau-b (τ_b_) correlation coefficients and its p-values were calculated. BDNF, brain-derived neurotrophic factor; n_pairs_, number of pairs analyzed. The level of significance was set at p < 0.05. Significant correlations are in bold italics.*

## Discussion

The present study investigated the effects of both acute (35 min) and prolonged (12 weeks) CE and PE alone and in combination on BDNF concentrations in plasma and serum in older adults. Associations between baseline pBDNF and sBDNF, between changes in pBDNF and sBDNF in response to acute and prolonged exercise, and between changes in CRF and BDNF from pretest to posttest were also assessed. The main finding of the present study was that even though a weak positive correlation was found between baseline BDNF in plasma and in serum, pBDNF and sBDNF concentrations responded differently to both acute and prolonged exercise in older adults. Different patterns of changes in pBDNF and sBDNF in response to exercise were confirmed by non-significant associations between changes, both acute and in response to prolonged exercise, in pBDNF and sBDNF in all of the investigated groups. Acute CE or PE induced a similar degree of increase in pBDNF in all of the investigated groups, even after 35 min of rest in the CE and PE groups. For sBDNF however, a significant acute increase in BDNF was shown only after PE in the three groups performing PE. Moreover, after 35 min of rest in the PE group, sBDNF levels returned to baseline levels, whereas in the CE group, sBDNF significantly decreased to below baseline levels after 35 min of rest. In the older adults studied, no significant correlation was found between baseline CRF and pBDNF or sBDNF, or between changes in fitness and changes in pBDNF or sBDNF, from pretest to posttest.

### Association Between Baseline Plasma and Serum BDNF

Evidence of positive associations between central and peripheral BDNF in plasma has been shown in previous research (Pillai et al., 2010; Klein et al., 2011). As it has previously been reported that BDNF can cross the human BBB (Pan et al., 1998; Krabbe et al., 2007), increased levels of peripheral BDNF are a potential indicator of elevated secretion of central BDNF (Krabbe et al., 2007). In the present study, a highly significant but rather weak positive correlation (*r* = 0.41) was found between baseline levels of pBDNF and sBDNF (Figure 4), in line with a previous study (Kudlek Mikulic et al., 2017). Inconsistencies in the strength or significance of a correlation between baseline pBDNF and sBDNF reported in the literature (Bocchio-Chiavetto et al., 2010; Yoshimura et al., 2010; Kudlek Mikulic et al., 2017; Polyakova et al., 2017) may be explained by the difference in investigated populations and the use of different anticoagulants for the pBDNF samples. For example, it has been shown that age may have an impact on pBDNF levels [pBDNF decreases with increasing age (Lommatzsch et al., 2005)] and gender may have an impact on sBDNF levels [lower sBDNF in women compared to men (Lommatzsch et al., 2005)], and that use of different anticoagulants and pre-analysis storage conditions for plasma such as storage time and temperature may affect BDNF concentrations in plasma (Tsuchimine et al., 2014).

The positive association between baseline pBDNF and sBDNF shown in the present study may not always be present since the main sources, biosynthesis and the physiological roles of BDNF are likely different in plasma and serum (Fujimura et al., 2002; Lommatzsch et al., 2005). For example, the potential sources of BDNF in plasma (circulating BDNF) are neurons and other cells of the CNS (Pan et al., 1998), vascular endothelial (Nakahashi et al., 2000) and vascular smooth muscle (Donovan et al., 1995) cells, lymphocytes (Sobue et al., 1998), and others mentioned in a previous review (Fujimura et al., 2002). It has also been suggested that the brain is a major source of resting pBDNF, as it has been shown that the brain contributes to the circulating BDNF in plasma at rest by 70–80% (Rasmussen et al., 2009). The BDNF in serum, however, is mainly stored in platelets circulating in human blood (Yamamoto and Gurney, 1990; Fujimura et al., 2002). As shown in previous research (Rosenfeld et al., 1995; Radka et al., 1996), and in the present study, BDNF levels in serum were on average approximately 100-fold higher than in plasma. Levels of BDNF in serum were significantly higher even if compared to BDNF levels found in the brain (Barde et al., 1982). The BDNF bound in platelets can be released into the bloodstream. Understanding to what extent such a transfer of BDNF from platelets to plasma occurs as a response to CE or PE is vital in the understanding of the effects of exercise on neuroplastic signaling.

### Association Between the Effects of Exercise on Plasma Versus Serum BDNF

#### Effects of Acute Exercise

Even though the majority of studies investigated the effects of acute PE on BDNF measured in serum, a significant increase in both pBDNF and sBDNF after acute PE has been shown in previous meta-analysis (Dinoff et al., 2017). The present study is one of the first studies to report the effects of PE on both pBDNF and sBDNF, as only 4 out of 55 studies reviewed in Dinoff et al. (2017) measured BDNF in both plasma and serum. However, these studies mainly investigated younger populations (mean age: 27.9 ± 10.8) (Dinoff et al., 2017).

According to the results of the present study, acute exercise interventions affected BDNF concentrations differently in plasma and serum (Figures 3A,B). There was also no significant association between acute changes in pBDNF and acute changes in sBDNF in response to CE and PE at pretest (Table 3). A significant gradual increase in pBDNF acutely after both the first and second exercise (or rest) was shown in all of the investigated groups (CE, PE, CE + PE, and PE + CE) at both pre- and posttest (Figure 3A). In serum, however, a significant increase in BDNF was seen only after acute PE in the PE, CE + PE and PE + CE groups, similarly at both pre- and posttest. Moreover, no significant change in sBDNF was shown after CE in the present study (Figure 3B). These results may suggest that acute PE has a significant positive effect on both pBDNF and sBDNF, whereas acute CE seems to only induce change in pBDNF. However, a recent study in older adults has reported an increase in BDNF in serum but not in plasma in an acute response to 40 min of moderate aerobic exercise (Máderová et al., 2019). The finding that changes in pBDNF do not always follow changes in sBDNF lends some support to the idea that the relationship between BDNF bound in platelets and BDNF in the bloodstream can change with CE or PE. Moreover, a passive control group (with no intervention prescribed) was not included in the present study and therefore, some effect of non-physical stress on changes in BDNF due to the test situation itself cannot be excluded.

A recent animal study reported evidence for the notion that lactate produced during PE is able to cross the BBB, and induces hippocampal BDNF expression and signaling that in turn enhances learning and memory (El Hayek et al., 2019). A positive association has also been shown between changes in blood lactate and changes in sBDNF from baseline to post-exercise following a graded exercise test on a stationary cycle ergometer (*r* = 0.57; *p* < 0.05) (Ferris et al., 2007) and hypertrophy resistance exercise (*r* = 0.70; *p* < 0.01) (Marston et al., 2017). Moreover, another study has shown that the brain contributes to BDNF concentrations in plasma during PE by 70–80% (Rasmussen et al., 2009). Therefore, the elevated pBDNF concentrations after both CE and PE shown in the present study may indicate an elevated BDNF secretion in the brain, which may in turn support neuroplasticity.

However, it is still uncertain whether an increased level of BDNF measured in serum is a valid indicator of an elevated secretion of BDNF in the brain (Chacón-Fernández et al., 2016; Naegelin et al., 2018). One of the potential sources of elevated concentrations of sBDNF in response to acute PE has been suggested to be increased platelet count in the bloodstream (Matthews et al., 2009; Walsh et al., 2017; Naegelin et al., 2018). A significant positive correlation between BDNF levels measured in serum and platelet count was demonstrated in a sample of 259 adults (Naegelin et al., 2018). In addition, two previous studies found a relatively close match between an increase in platelet count and sBDNF levels induced by PE (Matthews et al., 2009; Walsh et al., 2017). Interestingly, while Matthews et al. (2009) found an increase in sBDNF and platelet count after 120 min of bicycle exercise, i.e., whole body exercise, Walsh et al. (2017) found a significant effect from exercise involving only small muscle groups, specifically 10 min of maximal and 30 min of submaximal forearm handgrip exercise. Based on the results of their study, Walsh et al. (2017) suggested that as a result of PE, 30% of platelets may be stored in the spleen and that this may have significantly contributed to the elevated levels of sBDNF. PE may induce a sympathetic outflow of catecholamines such as epinephrine and/or norepinephrine which in turn would cause splenic constriction and stimulate thrombocytosis, i.e., the release of platelets stored in the spleen (Walsh et al., 2017; Walsh and Tschakovsky, 2018). Other potential sources for the increase in sBDNF due to PE may be the augmentation of platelet activation and reactivity (Kestin et al., 1993), and the ability of platelets to bind and internalize BDNF from other sources in the blood circulatory system (Fujimura et al., 2002; Lommatzsch et al., 2005) which in turn may also lead to an increased level of BDNF per platelet as found for example in previous study (Walsh et al., 2017).

Furthermore, platelets, once activated, by, for example, shear stress stimulation induced by PE (Fujimura et al., 2002), can release BDNF into the blood. Therefore, platelets may be an additional contributor to the elevated pBDNF during PE, and the released BDNF may induce neuroplastic changes in the periphery or even reach the brain via the BBB (Fujimura et al., 2002). Moreover, Matthews et al. (2009) showed that levels of BDNF in skeletal muscle are increased following exercise, illustrating an additional potential contributor to elevated levels of pBDNF post exercise (Matthews et al., 2009).

To our knowledge, the present study is the first study showing an increase in BDNF in plasma after acute CE alone (Figure 3A). This result may possibly indicate that the increase in pBDNF was a result of cognitive stimulation itself. However, we cannot exclude the possibility of a stress-related neuroprotective effect (Tapia-Arancibia et al., 2004; Rothman and Mattson, 2013). Co-regulation between the expression of cortisol and pBDNF has been hypothesized in a previous study (Begliuomini et al., 2008). Though analysis of cortisol was beyond the scope of the present study, cortisol levels from plasma samples collected during the present study will be assessed in a future study.

Another interesting result shown in the present study was that pBDNF levels did not return to baseline levels even after 35 min of rest in the CE and PE groups (Figure 3A). This result may be explained by elevated stress after exercise. However, we suggest that a recovery longer than 35 min post exercise is needed for pBDNF to return to baseline levels, as has been shown in a previous study (Rasmussen et al., 2009). In serum, however, BDNF had returned to baseline levels after 35 min of rest in the PE group (Figure 3B). These results may suggest that the effect of acute PE on sBDNF is a relatively short-term effect, which is in line with, for example, the results in another study that found sBDNF levels returned to baseline levels already after 15 min of recovery following an incremental maximal cycling exercise test (Rojas Vega et al., 2006). These results may solely be caused by a fast gradual decrease in platelet count in the circulating blood via the reuptake of platelets by the spleen as suggested in a previous review (Walsh and Tschakovsky, 2018). However, we could also hypothesize, and this seems to be more likely, that BDNF stored prior to exercise, and potentially sequestered from circulation (Fujimura et al., 2002) during exercise was released into the bloodstream together with platelet reuptake by the spleen during recovery. This process may also be somewhat reflected in the increased pBDNF concentrations seen even after recovery from PE in the present study (Figure 3A). However, as the decrease in sBDNF (∼5098 pg/mL average decrease) in the PE group (Figure 3B) was much larger than the increase shown in plasma (∼115 pg/mL average increase), we might also suggest that the BDNF released from platelets during recovery is quickly sequestered by other tissues in the circulation and possibly by neural cells in the brain after a possible crossing of the BBB.

#### Effects of Prolonged Training

In the present study, the 12 weeks of CE and PE, both alone and combined, did not induce a significant increase in baseline levels of BDNF in either plasma (Figure 3A) or serum (Figure 3B).

Only 31% of studies reviewed in a previous meta-analysis reported a significant elevation of basal levels of either pBDNF or sBDNF after prolonged exercise intervention (Dinoff et al., 2016). Furthermore, the majority of studies reviewed investigated younger populations and the reported results were also somewhat heterogeneous (Dinoff et al., 2016). In older adults, there is insufficient evidence and inconsistent results on the effects of prolonged PE intervention on peripheral BDNF. For example, no significant changes were found in either sBDNF after 1 year of prolonged aerobic exercise training (Erickson et al., 2011), or in pBDNF or sBDNF in response to 3 months of aerobic-strength exercise training (Máderová et al., 2019). However, an increase in pBNDF was found in older women in response to 16-week multimodal exercise training (Vaughan et al., 2014), and in a mixed gender group of older adults in response to 6 months of dance training (Rehfeld et al., 2018). Thus, based on the literature and the results of the present study we could speculate that either the effects of long-term exercise on baseline pBDNF and sBDNF concentrations are statistically negligible, or that a longer or more intense training period is required in older adults. However, Erickson et al. (2011) found a significant (*p* < 0.01) weak positive correlation between greater increases in sBDNF and greater increases in left and right hippocampal volume (*r* = 0.36 and *r* = 0.37, respectively). Furthermore, Erickson et al. (2011) also reported a significant positive relationship between an increase in hippocampal volume and an improvement in memory performance. While such a relationship between the effects of PE on peripheral BDNF levels and long-term improvements in cognition have sometimes been found (Erickson et al., 2011; Leckie et al., 2014; Vaughan et al., 2014; Rehfeld et al., 2018; Nilsson et al., 2020), there is low consistency between studies and further clarification is needed to understand how exercise-induced changes in peripheral BDNF affects neuroplasticity.

A significant (*p* < 0.05) decrease in pBDNF irrespective of the effect of “*time*” was shown in groups PE and CE + PE at posttest compared to pretest (Figure 3A). We could speculate that these results may potentially indicate that peripheral adaptation to cycling exercise reduces muscle expression of potential metabolic mediators important for BDNF signaling.

Finally, there was no significant association between changes in pBDNF and changes in sBDNF in response to the 12-week training (Table 3). This may indicate that the patterns of changes in pBDNF versus changes in sBDNF in response to 12 weeks of cognitive and physical training were also explicitly different in the investigated older adults.

### Association Between Cardiorespiratory Fitness and BDNF

In the present study, baseline CRF (VO2peak_*pre*_) did not correlate with baseline ln-pBDNF_*S*1_ and sBDNF_*S*1_ (see section “Association Between Cardiorespiratory Fitness and BDNF”). The previously reported inverse association between baseline CRF and baseline sBDNF (Currie et al., 2009; Jung et al., 2011; Hwang et al., 2017) was not confirmed in the older adults investigated in the present study. This may indicate that the association between CRF and BDNF is not present in older adults. However, another possible explanation for the non-significant association shown in the present study could be that even though participants did show variation in VO2peak_*pre*_ [range (min − max) = 19.4–47.5 mL kg^–1^ min^–1^; SD = 5.5 mL kg^–1^ min^–1^], approximately 81% of the participants ranged in fitness between 24 and 38 mL kg^–1^ min^–1^. We may speculate that a population with a larger and more evenly spread variation in CRF is needed to be able to detect a significant association between baseline fitness and baseline BDNF. For example, 865 participants older than 30 years in Jung et al. (2011) exhibited a variation in CRF between approximately 20 and 60 mL kg^–1^ min^–1^.

Physical exercise did not result in a change in CRF. We therefore cannot exclude the possibility that a higher variation in CRF or exercise at a higher intensity or a longer period would have been required to investigate a possible association between changes in fitness and changes in BDNF in older adults.

### Limitations

There are some limitations that may have affected the results of the present study and possibly their interpretation. Concerning the levels of CRF in the investigated population, the variation in fitness levels was rather small. We speculate that we would possibly find some association between baseline CRF and BDNF as seen in the literature if we had chosen a population with a larger variation in fitness. Even though measurements of stress, such as cortisol, were beyond the scope of the present study, such analysis would improve the interpretation of BNDF changes seen after exercise. For example, cortisol could have been used in the analysis as a covariate in order to control for psychosocial stress factors. Another limitation of the present study may be the lack of a passive control group (with no intervention prescribed). The inclusion of such a control group could allow for a more meaningful assessment of the effects of different interventions on BDNF levels, and give an opportunity to explore the potential effect of non-physical stress on changes in BDNF due to the test situation itself. In future, a study with a crossover design, preferably with a more mechanistic approach, should be considered to better clarify the physiological roles of and the association between pBDNF and sBDNF, and their responses to different types of exercise. In the current study, we kept the relative exercise intensity constant between pre and post the 12-week training. The absolute loads of both the PE and CE were therefore increased. Despite this increase in absolute load after the intervention period (due to increased PE and CE capacity), we did not find any increase in BDNF. We cannot exclude the possibility that we would have found a decrease in acute effects of PE and CE on BDNF if we had kept the absolute rather than the relative loads constant between the pre and post training period.

## Conclusion

The results of the present study clearly show that CE and PE may both elevate peripheral BDNF concentrations in older adults. Furthermore, while a significant weak positive association was found between baseline pBDNF and sBDNF, the CE and PE affected plasma and serum BDNF concentrations in different ways. Based on previous research and the results of the present study, we suggest that until the association between pBDNF and sBDNF, as well as the source and physiological roles of BDNF in both plasma and serum is thoroughly scientifically clarified, future studies should assess BDNF concentrations in both plasma and serum. This study did not provide evidence for the association between CRF and BDNF in older adults. However, we suggest that future studies should consider a population with a larger and more evenly spread variation in CRF for further investigations of this relationship in older adults.

## Data Availability Statement

The datasets presented in this article are not readily available because Swedish data protection laws prohibit publishing these data in the public domain. Requests to access the datasets should be directed to the authors. Upon request, the datasets may only be transferred for well-defined analysis projects that are in line with the original ethics approval. A data use agreement is required. This agreement will effectively transfer the confidentiality obligations of the institution (Karolinska Institutet) at which the original research was conducted to the institution receiving the data. Requests to access the datasets should be directed to OT, olga.tarassova@gih.se.

## Ethics Statement

The studies involving human participants were reviewed and approved by the ethical review board in Stockholm (Regionala Etikprövningsnämnden, Stockholm, case number 2017/1115-31/4). The patients/participants provided their written informed consent to participate in this study.

## Author Contributions

ML contributed to the conception of the study. JN and ME were responsible for the study design. ME, MM, and OT contributed to the data acquisition and analysis. OT performed the statistical analysis and wrote the first draft of the manuscript. All authors contributed to manuscript revisions, and read and approved the submitted version.

## Conflict of Interest

The authors declare that the research was conducted in the absence of any commercial or financial relationships that could be construed as a potential conflict of interest.
