# Supplementary material for: Peripheral BDNF Response to Physical and Cognitive Exercise and Its Association With Cardiorespiratory Fitness in Healthy Older Adults
Source: Front Physiol. 2020 Aug 25;11:1080. doi: 10.3389/fphys.2020.01080 (PMC7477111; doi:10.3389/fphys.2020.01080)
Supplement: Supplementary file 1 [file Table_1.DOCX]

Supplementary Material

# Supplementary Tables

**Supplementary Table 1.** Descriptive statistics for non-transformed plasma BDNF (pBDNF) and serum BDNF (sBDNF) measured at pretest from blood sample: S1 (baseline), S2 (acutely after first exercise) and S3 (acutely after second exercise or rest) and at posttest from blood sample: S4 (baseline), S5 (acutely after first exercise) and S6 (acutely after second exercise or rest) for groups that performed either cognitive only followed by rest (CE), physical only followed by rest (PE), a cognitive followed by physical (CE+PE) or a physical followed by cognitive (PE+CE) exercise. Concentrations for pBDNF and sBDNF are presented as mean ± standard deviation (SD). BDNF: Brain-derived Neurotrophic Factor; n: number of participants.

|  | **CE** | | | **PE** | | | **CE+PE** | | | **PE+CE** | | |
| --- | --- | --- | --- | --- | --- | --- | --- | --- | --- | --- | --- | --- |
|  | n | mean | SD | n | mean | SD | n | mean | SD | n | mean | SD |
| **pBDNF (pg/mL)** |  |  |  |  |  |  |  |  |  |  |  |  |
| **pretest** |  |  |  |  |  |  |  |  |  |  |  |  |
| **S1** | 21 | 189 | 152 | 27 | 389 | 507 | 23 | 238 | 205 | 25 | 198 | 211 |
| **S2** | 21 | 1100 | 1904 | 27 | 1463 | 2045 | 24 | 1663 | 3536 | 25 | 684 | 844 |
| **S3** | 21 | 1358 | 1730 | 27 | 1770 | 2767 | 24 | 2709 | 3805 | 25 | 1270 | 2025 |
| **posttest** |  |  |  |  |  |  |  |  |  |  |  |  |
| **S4** | 21 | 363 | 493 | 24 | 163 | 130 | 22 | 228 | 175 | 23 | 225 | 201 |
| **S5** | 21 | 1098 | 2658 | 25 | 701 | 1093 | 22 | 437 | 414 | 23 | 510 | 532 |
| **S6** | 21 | 1596 | 2847 | 25 | 842 | 974 | 22 | 1116 | 2286 | 23 | 1056 | 2094 |
| **sBDNF (pg/mL)** |  |  |  |  |  |  |  |  |  |  |  |  |
| **pretest** |  |  |  |  |  |  |  |  |  |  |  |  |
| **S1** | 20 | 25011 | 4958 | 26 | 23964 | 4979 | 23 | 25696 | 7077 | 25 | 24198 | 5336 |
| **S2** | 20 | 25238 | 4510 | 26 | 28244 | 6365 | 24 | 26635 | 6116 | 25 | 28314 | 6803 |
| **S3** | 20 | 22730 | 7890 | 26 | 23726 | 6785 | 24 | 29185 | 7680 | 25 | 24616 | 7358 |
| **posttest** |  |  |  |  |  |  |  |  |  |  |  |  |
| **S4** | 20 | 25865 | 4433 | 25 | 25244 | 6424 | 22 | 27981 | 6189 | 23 | 25738 | 5382 |
| **S5** | 20 | 26347 | 4694 | 25 | 29463 | 7224 | 22 | 26878 | 5231 | 23 | 28582 | 4251 |
| **S6** | 20 | 23589 | 5653 | 25 | 23785 | 6108 | 22 | 30388 | 6386 | 23 | 25020 | 3648 |
